# Canada: will privacy rules continue to favour open science?

**DOI:** 10.1007/s00439-018-1905-0

**Published:** 2018-07-16

**Authors:** Adrian Thorogood

**Affiliations:** BCL/LLB, Centre of Genomics and Policy, McGill University, Montreal, Canada

## Abstract

Canada’s regulatory frameworks governing privacy and research are generally permissive of genomic data sharing, though they may soon be tightened in response to public concerns over commercial data handling practices and the strengthening of influential European privacy laws. Regulation can seem complex and uncertain, in part because of the constitutional division of power between federal and provincial governments over both privacy and health care. Broad consent is commonly practiced in genomic research, but without explicit regulatory recognition, it is often scrutinized by research or privacy oversight bodies. Secondary use of health-care data is legally permissible under limited circumstances. A new federal law prohibits genetic discrimination, but is subject to a constitutional challenge. Privacy laws require security safeguards proportionate to the data sensitivity, including breach notification. Special categories of data are not defined a priori. With some exceptions, Canadian researchers are permitted to share personal information internationally but are held accountable for safeguarding the privacy and security of these data. Cloud computing to store and share large scale data sets is permitted, if shared responsibilities for access, responsible use, and security are carefully articulated. For the moment, Canada’s commercial sector is recognized as “adequate” by Europe, facilitating import of European data. Maintaining adequacy status under the new European General Data Protection Regulation (GDPR) is a concern because of Canada’s weaker individual rights, privacy protections, and regulatory enforcement. Researchers must stay attuned to shifting international and national regulations to ensure a sustainable future for responsible genomic data sharing.

## Introduction

Canada’s research funding agencies actively promote open science. Genomic data sharing is practiced widely, and has yet to encounter an existential conflict with regulatory frameworks governing privacy and research. Data sharing may, however, attract greater regulatory scrutiny following the recent overhaul of European privacy laws. Data sharing is the research practice of making individual-level human genomic and health-related data used in studies available to other scientists. It is often achieved by depositing data supporting publications in community databases. Major funding agencies have open access policies that encourage or require data sharing (e.g., Genome Canada 2016; Canadian Institutes of Health Research 2017). Across the health sciences, however, the practice of data sharing remains limited (Stuart et al. 2018), due in part to a lack of data sharing requirements; insufficient financial support, incentives, infrastructure, data standards, expertise; and—the focus of this article—real or perceived legal and regulatory barriers (Council of Canadian Academies 2015). Data sharing also refers to the practice of providing researchers access to both genomic and clinical data generated in health-care contexts. Some predict that tens of millions of genomes may be sequenced globally for health-care purposes by 2020, presenting major opportunities for research (GA4GH 2017).

Numerous publicly funded data sharing “platforms” have been established to enable Canadian researchers to collaborate and share computing resources, analysis tools, and research data. National genomic data sharing initiatives include CanDIG (2018), Care4Rare SOLVE (2018), the Google/MSSNG autism database (2018), the Personal Genome Project Canada (Reuter et al. 2018), and the Canadian Open Neuroscience Platform (2018). These initiatives are complemented by national biobanking efforts, including the Canadian Partnership for Tomorrow Project (2018). Storing, analysing and sharing large genomic data sets are increasingly synonymous with cloud computing solutions and services (Thorogood et al. 2016). Canada also plays a leadership role in the science and governance of international data sharing initiatives such as the International Cancer Genome Consortium (ICGC) http://icgc.org, and the International Human Epigenome Consortium (IHEC) http://ihec-epigenomes.org. Large, publicly funded provincial health systems present rich and comprehensive data sources for research and linkage with genomic data. Provincial governments are interested in making more effective research use of electronic medical record data (e.g., Ontario Genomics 2015; Québec INESS 2018). National approaches to data governance and access, however, are likely to remain “a dream” (Morin and Flegel 2017).

Genomic data sharing raises ethical and legal concerns over participant and patient privacy. Genomic data is unique to the individual, and contains latent, sensitive health and other information about individuals and their families (NHGRI 2015). Researchers also wish to combine genomic data with an expanding range of clinical, mobile, and environmental data to identify patterns linking genetic variation with disease and treatment response. Big Data analytics and Artificial Intelligence promise to revolutionize research and health care, but also make data identifiability and sensitivity moving targets (Schadt 2012). Persistent privacy concerns include employer, insurer and other forms of discrimination (Joly et al. 2017); law enforcement access (Ram et al. 2018); and research uses that do not serve the public interest or respect participants’ expectations. Recent controversies have reinforced these concerns, such as the familial matching of the Golden State Killer using consumer genetic databases (Kolata and Murphy 2018), and the breach and misuse of Facebook data from millions of US and Canadians by Cambridge Analytica (Huncar 2018).

This article reviews the Canadian regulatory framework applying to data sharing, with a focus on issues of consent, privacy and security, international regulatory compatibility, and oversight. A simplified overview is provided here. A right to privacy is implicitly enshrined in Canada’s constitutional Charter of Rights and Freedoms (ss. 7 & 8) (Government of Canada 1982; Saulnier and Joly 2016). More directly applicable to data sharing are federal and provincial personal information protection statutes, applying variously to the private, public, and health sectors (for a detailed review, see Thorogood et al. 2016). These laws govern the collection, use, and disclosure of personal information. The federal private sector data protection law, PIPEDA, applies nationally as a baseline, but may be displaced by provincial private or health sector legislation deemed by the federal government to offer substantially similar protection (OPC 2017b). Research institutions receiving public funding are also governed by the Tri Council Policy Statement (TCPS2) national health research guidelines (2014).

Health care and privacy regulation are affected by the structure of Canada’s health-care system, which “is not a national system per se, but rather a collection of provincial and territorial health insurance plans subject to national standards” (Martin et al. 2018). The federal government provides significant federal funding to the provincial and territorial plans, provided they adhere to the *Canada Health Act* standards of portability, universality, accessibility, comprehensiveness, and public administration (Government of Canada 1985). Administration and delivery are highly decentralized (Martin et al. 2018). Provinces also have distinct personal (health) information protection laws and health-care data governance strategies. National coordination of privacy regulation is complicated by this nuanced and sometimes contested division of powers between federal and provincial governments.

Two external forces are exerting pressure for legal and regulatory reform in Canada. The first is rapid technological change. Cloud computing platforms, genomic sequencing, high resolution imaging, and mHealth apps enable unprecedented health data generation and sharing. The categories of “health-related” data are constantly expanding, as are the types of organisations involved in health research and health care. Big Data and Artificial Intelligence analytics present new opportunities for harnessing the mounting data deluge to improve health care, but raise new concerns over responsibility, validity, bias, and privacy (Nuffield Council on Bioethics 2018). Technological advances promise to revolutionize health research and health care, but may also fundamentally threaten the effectiveness of existing laws and regulatory frameworks (Brownsword et al. 2016). The second external pressure is the influential General Data Protection Regulation (GDPR), now in force across Europe (European Parliament and Council 2016). Canada has adopted European style privacy protections, formally recognized as “adequate” to protect European data transferred to Canada. To maintain its adequacy status, Canada may be expected to harmonize its framework with the GDPR’s strengthened privacy protections (House of Commons Hearings 2018). Canadian health researchers must remain attuned to the shifting winds of privacy regulation both nationally and globally.

## Consent

A basic principle of data protection law is that personal information must only be collected, used, or disclosed with the consent of the individual (or another legal basis). Consent must be meaningful, and can be withdrawn (revoked) at any time. Canadian law makers are exploring ways to strengthen consent requirements, so it can remain a central safeguard (House of Commons Report 2018). The key question here is what constitutes meaningful consent to genomic research and data sharing? Is a broad consent to sharing data with a wide research community for future not-fully specified research acceptable? Research and data sharing where consent has not been obtained are discussed below (“Oversight”).

Broad consent is commonly practiced in genomics research and biobanking in Canada (Allen et al, 2017). This is normally defined as consent to the future use of samples and data that are not fully specified in advance, combined with ongoing governance (CIOMS/WHO, 2016). Broad consent is indirectly supported by the Canadian legal doctrine of consistent use (Archibald and Lemmens 2008). Ethically, the TCPS2 guidelines recognize but do not fully condone broad consent, unlike international guidelines and the US Common Rule (CIOMS/WHO 2016; US Government 2017). The TCPS2 generally requires “full and frank disclosure” of all relevant information, including detailed information about handling of private information (TCPS2 2014, arts. 2.7 & 3.2). Consent requirements include detailed information about how data will be handled, though a research ethics board (REB) may waive or modify consent requirements where research is minimal risk, unlikely to adversely affect participant rights and welfare, transparent, and where consent is not practicable (TCPS2 2014, art. 2.1). Arguably, the lack of legal and policy clarity remains a source of confusion for REBs, which can lead to inconsistent decisions.

In Europe, the GDPR requires that consent to the processing of personal information be explicit, specific, clear, and not coerced, but explicitly recognizes that consent to research cannot always be fully specified in advance (European Parliament and Council 2016, art 5.1.b, rec. 33), though this may be interpreted narrowly (Article 29 Working Party 2018). A Canadian House of Commons Committee recently recommended the strengthening of consent under PIPEDA, including opt-in consent by default for secondary use (House of Commons Report 2018). While strengthening individual control, opt-in by default could have devastating consequences for researcher access to and use of data generated in health contexts (Council of the Canadian Academies 2015).

## Privacy

Canadian personal information protection laws apply to personal information, which is defined in broad terms as information that relates to an identifiable individual. The TCPS2 research ethics guidelines use the privacy categories identifiable, coded, anonymized, and anonymous, and call for researchers to establish appropriate privacy and security protections (Ch 5). Canada does not deterministically define special categories of data as “sensitive”. Instead, sensitivity is determined contextually on a case-by-case basis, and proportionate safeguards must be in place (PIPEDA). Health and genetic data are, however, generally considered sensitive by Canadian courts and regulators. Data minimization and purpose limitation principles are recognized in Canada, though these cut against Big Data approaches to health research. In response, privacy commissioners encourage privacy by design approaches, the proactive embedding of privacy into information technology, business practices, and networked infrastructures, to anticipate and prevent privacy invasive events before they occur (Cavoukian 2014).

Some forms of potential misuse of genomic data are now prohibited by law. Canada’s federal *Genetic Non-Discrimination Act* makes it a criminal offence to require disclosure of genetic test results as a condition of entering into a contract; or to collect, use or disclose genetic test results without explicit written consent (Government of Canada 2017). This should allow Canadians to seek genetic testing or participate in genomic research without fear of insurer or employer discrimination. The province of Quebec, however, is challenging the constitutionality of this law, arguing it infringes on provincial powers to regulate insurance (Bombard and Heim-Myers 2018). Uncertainty over the law’s constitutionality may reinforce persistent public skepticism over protections against the use of genetic data collected in clinical and research contexts for insurance purposes (Joly et al. 2017).

The EU right to be forgotten encompasses a right to erasure of personal information (from a website), as well as a right to de-indexing from a search engine. It is unclear if this right is fully recognized in Canada, or if it should be. The OPC draft guideline suggests that PIPEDA already includes a right to have personal information erased or de-indexed, though the feasibility of this right in digital environments is questioned (OPC 2018). The Quebec Privacy Commissioner, by contrast, found no basis for such a right in a recent case about de-indexing of a former employee profile (C.L. v. BCF 2016). If recognized, would this right extend to the withdrawal of data from genomic research projects and databases?

Another emerging privacy question for health researchers is whether or not they are legally required to provide participants’ access to their data, upon request (Thorogood et al. 2017). The EU GDPR includes a general right to access one’s personal information, though nation states may implement legal exceptions in the research context (European Parliament and Council 2016, arts. 15 & 89). Canadian law recognizes broad rights to access one’s health record (McInerney v. MacDonald 1992), and one’s personal information held by public or private sector organizations, though (narrow?) exceptions are typically made in research contexts (Ries 2010). Considering the EU GDPR, widespread data sharing between researchers, patient empowerment movements, and a shift towards translational research based in clinical contexts, participant access to raw sequence data may become an increasingly contentious policy and legal issue in Canada.

## Security measures

More data collection, access, sharing, and use tends to give rise to commensurate security problems. In recent years, private and health sector organizations have experienced a number of high profile security breaches. In 2013, an unencrypted laptop was stolen from a network of medical centres in Alberta. Affecting 620,000 patients, the breach led to a six figure class action settlement (Mah 2016). In Ontario, private information from thousands of new mothers held by the Rouge Valley Health System was stolen by employees and sold to financial firms peddling education savings plans (CBC News 2015). Individuals involved were criminally charged, a 400$ million class action was launched, and the provincial privacy commissioner found the hospital failed to adequately restrict employee searches. Moreover, the two breaches resulted in legislative backlash, with both provinces increasing regulatory fines for security breaches (Mah 2016; CBC News 2015). Despite this weak track record, Canadian privacy statutes do not include detailed security requirements. The federal privacy sector law PIPEDA simply requires custodians of personal information to establish physical, organizational, and technological measures appropriate for the sensitivity of the information (Government of Canada 2000, Schedule 1, s. 4.7). In the absence of detailed legal rules or interpretations, Canada’s open science community is likely to look to international security standards (e.g., ISO, NIST). Custodians are generally permitted to transmit data to a (cloud) service provider if uses are appropriate limited and security is ensured through contract. The technical, organizational, and cross-border complexity of the cloud, however, raises uncertainty about who can access data, what data might be used for, and how security responsibilities are shared (Thorogood et al. 2016). In response, cloud service providers are localizing servers in Canada and tailoring offerings to the Canadian regulatory context (e.g., Canadian Genomics Cloud 2018).

Mandatory breach notification provisions were added to PIPEDA under the *Digital Privacy Act*, and come into force in late 2018 (Government of Canada 2018). Custodians are required to keep records of security breaches involving personal information, and to notify both the federal privacy regulator and the affected individuals of all breaches where it is reasonable to believe that the breach creates a real risk of significant harm. Notice must be given in sufficient detail and as soon as it is feasible. Reporting provisions encourage risk mitigation and improve transparency and accountability for security practices. Presumably custodians are required to extend this requirement to cloud service providers under the accountability principle (Cameron and Feltrin 2017). Adoption of breach notification provisions brings Canada in line with international standards (OECD 2013; European Parliament and Council 2016). Identifying breaches may, however, be both conceptually and practically challenging for Canada’s open science community. What types of research data are personally identifiable? Where data are consented for wide sharing and reuse, can “breach” be clearly defined? Could the risks of a breach meet the high standard required for reporting? Researchers may also need to consider incorporating breach notification provisions into cloud service provider and data sharing agreements.

## Compatible processing/adequacy

Under what conditions can Canadian researchers transfer rich individual-level data to collaborators in foreign countries? PIPEDA holds custodians accountable for data transferred to third parties. Under this accountability model, custodians are generally permitted to transfer personal information outside Canada, as long as they establish appropriate contractual or other safeguards. A slightly stronger accountability standard is imposed under Quebec’s private sector law, which provides that custodians must take all reasonable steps to ensure that the data will not be used for unrelated purposes without the consent of the persons concerned (Government of Quebec 1991, s. 17). In Canada, the burden is on custodians to determine if foreign laws provide appropriate legal protections. The difficulty for research and health-care institutions to make such assessments is reflected by increased data residency offerings by cloud service providers (e.g., Canadian Genomics Cloud 2018). Exceptionally, the provinces of British Columbia and Nova Scotia prohibit the transfer of personal information held by public sector organizations outside the province except with the explicit consent of the individual (Government of British Columbia 1996, s. 33.2; Government of Nova Scotia 2006, s. 5(1)). These data residency requirements are motivated by concerns over mass law enforcement surveillance in the United States. Canada has entered into or is negotiating a number of international trade agreements that incorporate the principle of open data flows (e.g., TPP, CETA, NAFTA), which may affect the validity of provincial data residency laws.

In what circumstances can foreign collaborators, particularly Europeans, transfer data to Canada? Under the European adequacy approach, the EU Data Protection Board, rather than individual organisations, assesses the adequacy of foreign legal frameworks (European Commission 2018; European Parliament and Council 2016, art 45). Canada’s commercial sector under PIPEDA is currently considered adequate, though it is somewhat unclear if it will remain so under the new GDPR. Adequacy considerations include assessment of the rule of law, legislation and its implementation, access by public authorities, and the functioning and powers of a supervisory authority (European Parliament and Council 2016, art 45). Assessment may be granted to a specific territory or sector within a country. Areas where PIPEDA may be found to fall short of the strengthened GDPR protections include a lack of (explicit) definition of sensitive categories of data (and related protections), the right to be forgotten, the right of data portability, data protection by design, and the enforcement powers of the federal privacy regulator (House of Commons Report 2018). Canada’s adequacy will come up periodically for review, or might even be challenged in court. Simply updating the language of PIPEDA may be insufficient. Case law indicates that adequacy assessments may look beyond black letter laws governing personal data transactions, to consider a state’s broader data governance and law enforcement surveillance practices. In Schrems, the Court of Justice of the EU case that struck down the US-EU Safe Harbour agreement on data transfers because of concerns about mass surveillance (CJEU 2015). In API/PNR, the CJEU’s scrutinized an air passenger information sharing agreement between the EU and Canada, and highlighted concerns about protection of sensitive categories of data, and restricting Canada’s onward sharing of data with foreign authorities (CJEU 2017).

A future adequacy decision may also scrutinize the strength of provincial privacy laws. Indeed, controversy arose when the EU (still under the former Directive) questioned the adequacy of Quebec’s provincial framework, despite the Canadian requirement that provincial laws only displace PIPEDA if the federal government determines they are “substantially similar” (Stoddart et al. 2016). If PIPEDA alone is strengthened, provincial personal information laws may need to be updated in turn. Such coordinated legislative action can be politically and practically challenging in Canada.

## Oversight

Federal and provincial privacy commissioners oversee the privacy and security practices of organizations dealing with personal (health) information in Canada. The federal Office of the Privacy Commissioner (OPC) investigates complaints and mediates compliance agreements with custodians under PIPEDA (Government of Canada 2000, s. 12). As an “ombudsman”, the OPC cannot initiate its own investigations, administer fines or make binding orders without the involvement of the courts. Some provincial privacy commissioners, by contrast, have fining and order making powers (OPC 2017a). Numerous commentators and reports have called for expanding the OPC’s powers, in part to maintain “adequacy” vis-a-vis the EU GDPR (House of Commons Report 2018). There is also demand for a new individual statutory cause of action to pursue custodians directly for damages. Canadian researchers accessing data from Europe should also be aware of the potential extra-territorial application of the GDPR, with its eye catching fines (European Parliament and Council 2016, art. 3).

REBs also play an important, legally enshrined, oversight role of data-intensive research. Their role is of particular importance where researchers seek to access and use personal (health) information in the absence of consent, or a sufficiently specific consent (Council of Canadian Academies 2015). This “secondary use” mechanism is key for genomics, where researchers are increasingly interested in access to molecular and clinical data generated in health-care contexts (GA4GH 2017). Privacy laws in Canada typically allow access to personal (health) information to researchers with an REB approved protocol, and who sign a confidentiality and security agreement. REBs, in turn, can waive consent requirements if identifiable information is essential to the research, appropriate measures are taken to protect privacy and confidentiality and to minimize harms to subjects, and the individuals have not objected (TCPS2 2014, art. 3.3). In Quebec, researcher access to health-care data may additionally require approvals from institutions and administrative bodies (Government of Quebec 1991, s 19.2).

Data access committees (DACs) complement REBs in overseeing researcher access to data. Canada has pioneered controlled access models to data sharing through the International Cancer Genome Consortium and the International Human Epigenome Consortium (Dyke et al. 2016). Following international data sharing guidelines, controlled access processes aim to balance between accessibility and protection of the rights of data generators and individual privacy. DACs may assess researchers’ qualifications, trustworthiness and protocols (Dyke et al. 2016). Approved researchers sign an access agreement, which usually includes commitments to limit use to the approved protocol, and to keep data confidential and secure. Canadian DACs typically require researchers to additionally undergo local ethics review before applying for access (e.g., Canadian Partnership for Tomorrow Project 2018).

## Future directions

Canada’s regulatory framework is relatively friendly to open science and international collaboration. There is pressure to strengthen laws and regulation in response to broader public worries over weak commercial data governance practices, and to align with European laws. Given the promise of genomics and Big Data to generate better health and more wealth, Canada may still decide to strike a distinct balance than the GDPR (Guilmain 2018). Explicitly defining “sensitive” categories of data, for example, entails numerous conceptual and practical difficulties (Hordern 2018). Beyond questions of legal compliance, ongoing engagement is to support the needs of scientists for more data and openness, while assuaging broader public worries over privacy. Indeed, public perceptions research continues to show limited societal support for broad consent approaches, intensified by growing support for perceived biorights (I “own” my data) and worries about commercialization and mishandling of health data (Caulfield and Murdoch 2017). The Canadian genomics community must continue to articulate a vision of the societal benefits of genomics data sharing for improving human health that convincingly outweighs these concerns.

There is also great enthusiasm that privacy-preserving technologies can cut the Gordian knot of privacy v.s. openness. Federated analysis, for example, allows researchers to run code on secure networks of datasets without accessing or copying the data (GA4GH 2016). Evolving technical safeguards such as differential privacy and encryption reduce the risks of re-identification and misuse without undermining access and utility (Erlich and Narayanan 2014). Sustained attention is needed to ensure the increasing technical complexity does not outpace the ability of regulators, researchers and citizens to engage with the societal implications of data sharing.
