# Correction to: Canada: will privacy rules continue to favour open science?

**DOI:** 10.1007/s00439-018-1922-z

**Published:** 2018-08-17

**Authors:** Adrian Thorogood

**Affiliations:** BCL/LLB, Centre of Genomics and Policy, McGill University, Montreal, Canada

## Correction to: Human Genetics 10.1007/s00439-018-1905-0

This article was inadvertently published under a draft title.

The final title for this article is the following:

"Canada: will privacy rules continue to favour open science?"

The article Canada: will privacy rules continue to favour open science?, written by Adrian Thorogood, was originally published electronically on the publisher’s internet portal (currently SpringerLink) on 16 July 2018 without open access. With the author(s)’ decision to opt for Open Choice the copyright of the article changed on 17 August 2018 to © The Author(s) 2018 and the article is forthwith distributed under the terms of the Creative Commons Attribution 4.0 International License (http://creativecommons.org/licenses/by/4.0/), which permits use, duplication, adaptation, distribution and reproduction in any medium or format, as long as you give appropriate credit to the original author(s) and the source, provide a link to the Creative Commons license and indicate if changes were made.

The original article was corrected.

